# Effectiveness-implementation hybrid designs: implications for quality improvement science

**DOI:** 10.1186/1748-5908-8-S1-S2

**Published:** 2013-04-19

**Authors:** Alice C Bernet, David E Willens, Mark S Bauer

**Affiliations:** 1 Veterans Affairs National Quality Scholars Program, VA Tennessee Valley Healthcare System, Nashville, Tennessee, 37212, USA; 2 Henry Ford Health System, Detroit, Michigan, 48202, USA; 3 Center for Organization, Leadership, and Management Research, VA Boston Healthcare System, Boston, Massachusetts, 02130, USA; 4 Department of Psychiatry, Harvard Medical School, Boston, Massachusetts, 02215, USA

## Presentation

This article summarizes the three types of hybrid effectiveness-implementation designs and associated evaluation methods. It includes a discussion of how hybrid designs have the potential to enhance knowledge development and application of clinical interventions and implementation strategies in “real world” settings. The authors propose implications of hybrid designs for quality improvement research.

Traditionally, researchers think of knowledge development and application as a uni-directional, step-wise, progression, in which different questions are addressed in isolation. First, a randomized clinical trial (RCT) is deployed to determine if an intervention implemented under controlled conditions has efficacy in specific populations. Next, “effectiveness research” methods determine if the effect remains when implemented in less controlled conditions with broader populations. Finally, “implementation research” methods, such as cluster randomized controlled trials, are deployed to understand the best methods to introduce the intervention into practice. While systematic, this unidirectional approach can take a great deal of time from the original efficacy study design to the final conclusions about implementation, and conditions may change so that original clinical and policy questions become less relevant [1]. Additionally, the unidirectional approach does not help us understand interaction effects between the intervention and the implementation strategy.

Hybrid designs simultaneously evaluate the impact of interventions introduced in real world settings (e.g. “effectiveness”), and the implementation strategy. Such designs enhance the ability to identify important intervention-implementation interactions, which inform decisions about optimal deployment and generalized impact, and may accelerate the introduction of valuable innovations into practice. This has implications for quality improvement researchers, who are often guiding the deployment and evaluating the impact of interventions in healthcare settings.

### Types of hybrid designs

Hybrid designs form a continuum between pure effectiveness research and pure implementation research, as defined above. Hybrid designs are best suited for the study of minimal risk interventions with at least indirect evidence of effectiveness, and strong face validity to support applicability to the new setting, population or delivery method in question. Each type describes two *a priori* aims: one for testing intervention effectiveness, and one for evaluating the implementation strategy. The types differ according to the emphasis placed on testing the intervention or the implementation. These designs incorporate evaluation methods—process, formative and summative evaluation—which distinguish hybrid designs from traditional effectiveness research but are typical of implementation research. For more detailed examples of hybrid design in published research, please refer to Curran et al. [2].

Type 1 hybrid designs rigorously test the clinical intervention and secondarily gather data to inform subsequent implementation research trials. These studies measure patient functioning or symptoms in response to a clinical intervention, while simultaneously evaluating feasibility and acceptability of implementation through qualitative, process-oriented, or mixed methods.

Type 2 hybrid designs simultaneously test the clinical intervention, while rigorously testing the implementation strategy. Ideal targets include populations or settings that are reasonably close to those studied in prior effectiveness trials. The use of fractional factorial designs can inform allocation of study groups in hybrid Type 2 studies. This design strategy allows for multiple “doses” of implementation resources to be available during a test of intervention effectiveness.

Type 3 designs primarily test the implementation strategy, via measures of adoption of and fidelity to clinical interventions. The secondary aim measures patient-level effects of the clinical intervention, such as symptoms, functioning and service use. When there is robust intervention data, but effects are suspected to be vulnerable during the implementation trial, a Type 3 design may elucidate the implementation barriers.

Process, formative, and summative evaluation methods are identical to those used in implementation research [3]. Process evaluation identifies potential and actual influences on the conduct and quality of implementation. In contrast to formative evaluation, data are not used during the study to influence the process. Data informing process evaluation can be collected prior to the study, concurrently, or retrospectively. Formative evaluation makes use of data throughout the intervention trial to modify the intervention procedures or implementation process *during* the study. To augment randomized or observational study designs, formative evaluation data are used in Type 2 and Type 3 hybrid designs to refine and improve the clinical intervention and implementation process while under study. This allows for real-time refinement of intervention and implementation techniques, however it may diminish external generalizability.

The summative evaluation provides information about the impact of the intervention, similar to classical clinical trials, which in this case of hybrid designs helps to inform a local healthcare system’s decision to adopt the intervention under study. The summative evaluation outcomes may include, for instance: patient level health outcomes for a clinical intervention, process or quality measures for an implementation strategy, population-level health status, or an index of system function for an organizational-level intervention. It is important to note that for hybrid designs, no less than for other types of studies, power considerations are important for summative evaluation outcomes both for intervention and the implementation measures.

## Commentary

Two aspects of hybrid designs are particularly germane to quality improvement research. One aspect is the summative evaluation. The summative evaluation provides additional contextual elements, which inform the decision of a healthcare system to adopt the intervention under study. Second is the formative evaluation aspect of hybrid designs. This type of evaluation is comparable to the Plan-Do-Study-Act (PSDA) methods used by quality improvement researchers to refine intervention and implementation strategies during the study. The rigor of formative evaluation and PDSA methods can be maximized when combined with robust study designs. Key features of these research designs include time series measurement, testing the stability of the baseline, use of replication and reversals, constancy of the treatment effect, and statistical techniques for evaluating effects [4].

Hybrid designs combine concepts that are familiar to improvement researchers, such as statistical methods to account for modifications to the intervention or improvement strategy, *a priori* considerations of process and clinical level outcomes, and a focus on interventions with at least indirect evidence of clinical effectiveness. As a result, the use of hybrid designs, and corresponding evaluation strategies, are primed for adoption by quality improvement researchers.

## Recommendations

Study of the contextual aspects of implementation and intervention effectiveness are essential to compare quality improvement interventions across a range of settings. Tools such as summative and formative evaluation could accomplish this. Hybrid designs would allow for evolving implementation and intervention strategies, which more closely resemble real-world changes to health care systems.

Grounding the use of hybrid designs in a unified conceptual framework will support the collaboration among professionals in quality improvement research, implementation science, and associated fields. Consideration for hybrid designs can further our understanding of how and why interventions may vary in effectiveness across settings. Such an achievement would enhance the legitimacy of quality improvement research as a solution to the problems facing healthcare.
